# Online training of Covid-19 infection prevention and control for healthcare workers in psychiatric institutes

**DOI:** 10.1186/s12888-023-04826-5

**Published:** 2023-05-09

**Authors:** Daiki Kobayashi, Kayama Mami, So Fujishiro, Noriaki Nukanobu, Shu-ichi Ueno, Shotaro Kuwakado, Tatsuya Koyama, Hironori Kuga

**Affiliations:** 1 Division of General Internal Medicine, Department of Medicine, Tokyo Medical University Ibaraki Medical Center, Ibaraki, Japan; 2 Fujita Health University, Toyoake, Japan; 3 Department of general medicine, Juntendo University Faculty of Medicine, Tokyo, Japan; 4 National College of Nursing, Tokyo, Japan; 5 Aichi prefectural mental health welfare center, Nagoya, Japan; 6 Faculty of Nursing, Hiroshima International University, Higashihiroshima, Japan; 7 Department of Neuropsychiatry, Ehime University Graduate School of Medicine, Matsuyama, Japan; 8 Medical Corporation Kifu-kai Association, Kawasaki, Japan; 9 Graduate School of Nursing, St. Luke’s International University, Tokyo, Japan; 10 National Center for Cognitive Behavior Therapy and Research, National Center of Neurology and Psychiatry, Kodaira, Japan

**Keywords:** Online, Training, Psychiatry, Covid-19, Japan, Infection prevention and control

## Abstract

**Background:**

This study aimed to develop a unique online infection prevention and control (IPC) training on Covid-19 for healthcare workers in psychiatric institutes in Japan and to examine its efficacy based on its impact on the knowledge, attitude, and confidence about IPC for Covid-19 among the healthcare workers.

**Method:**

This quasi-experimental study was conducted using online training on Covid-19 IPC for healthcare workers in various psychiatric institutes from April 2021 to March 2022. An online training video on Covid-19 IPC was developed. Voluntary healthcare workers in psychiatric institutes located in five prefectures in Japan were recruited to participate in this training. The participants then completed 30 min of online training and surveys about knowledge, attitude, and confidence were conducted pre, post, and three months after the training. The video training and surveys were contextually validated by the experts, but not by any previous study.

**Results:**

A total of 224 participants were included, of which 108 (54.0%) were men. The mean (standard deviation (SD)) age and the mean occupational experience were 47.4 (9.5) and 18.0 (12.6) years, respectively. Among the participants, 190 (84.8%) completed the post-training, and 131 (58.5%) completed the three-month-later training surveys. The total score on the quizzes in the post-training (+ 31.1%, SD 15.7, p-value < 0.01) and three-month-later training (+ 14.9%, SD 16.8, p-value < 0.01) surveys had significantly increased from that in the pre-training survey. In contrast, the total score in the three-month-later training had significantly decreased from that in the post-training survey (-16.1%, SD 16.7, p-value < 0.01).

**Conclusion:**

Thirty minutes of online training about IPC for Covid-19 had improved knowledge, confidence, and attitude among psychiatric healthcare workers. Regular online training would help in preventing the transmission or formation of clusters of Covid-19 in psychiatric healthcare institutes.

**Supplementary Information:**

The online version contains supplementary material available at 10.1186/s12888-023-04826-5.

## Introduction

Since Coronavirus disease 19 (Covid-19) outbreaks have been reported, most healthcare institutes, including the World Health Organization (WHO), [1] have provided infection prevention and control (IPC) training programs for their healthcare workers for both patient and staff safety. IPC is effective in reducing the transmission of Covid-19 not only between patients but also among healthcare professionals. Universal masking for healthcare professionals is one of the methods recommended to reduce the transmission, [2, 3] and is supported by previous studies which showed the decreased incidence of Covid-19 in healthcare facilities against the increased incidence in the communities [4, 5]. Similarly, hand hygiene is essential to reduce Covid-19 transmission in healthcare settings; however, compliance was limited even among healthcare professionals [6, 7]. Environmental cleaning [8] and zoning [9] are also important for Covid-19 IPC. Comprehensive strategies with these above methods, and not just one method, should be applied in healthcare facilities [10]. Thus, IPC is necessary to reduce the transmission of Covid-19 and all healthcare professionals should be trained and practice IPC measures.

However, few studies have evaluated the efficacy of the IPC program for Covid-19 targeting healthcare workers. Tadavarthy et al. developed and implemented an IPC program for physicians and nurses in a Covid-19 alternative care site in Philadelphia, [11] which resulted in improvement of patient and staff safety with the use of limited resources. A study from China developed and applied an online IPC program for pediatric healthcare workers, resulting in enhanced knowledge and awareness [12]. Another study from China developed a “four-step” mode of training and showed improvement in the staff’s level of IPC [13]. A group in Uganda has been challenged to develop a virtual reality-based IPC training for frontline healthcare workers [14]. Despite these studies, one research gap is that a limited number of previous studies provided and evaluated IPC training; additional studies to evaluate the efficacy of IPC training for specific healthcare workers are still required.

Another research gap from previous studies is that research about IPC for Covid-19 in psychiatric institutes is still lacking. IPC for Covid-19 in psychiatric institutes has been discussed to be challenging and different from other departments [15] as psychiatric patients were reported to have a higher risk of Covid-19 transmission, [16, 17] as well as higher Covid-19-related mortality [18]. Therefore, stricter IPC has been usually advocated for the psychiatric population such as performing Covid-19 tests for all admitted patients [19, 20]. In a psychiatric institute’s unique environment, patients were restricted to join group therapies or go to a common space, or were being treated by using indirect methods, such as online or phone calls [21]. Thus, special attention should be paid to Covid-19 prevention in psychiatric institutes and unique IPC training should be provided. However, studies providing IPC training and its evaluation in psychiatric institutes are very limited and desired intensely.

In this study, we aimed to develop a unique online IPC training on Covid-19 for healthcare workers in psychiatric institutes in Japan and to examine its efficacy on their knowledge, attitude, and confidence about IPC for Covid-19. One of the uniqueness of this study is that we provided individual and remote IPC training during the Covid-19 pandemic, which provided an opportunity for the healthcare workers to learn at their own pace without additional transmission risk. Also, the training was provided specifically for healthcare workers in psychiatric institutes where the risk of Covid-19 is higher. However, generalizability and transferability of the IPC training to other population may be less guaranteed in exchange for the uniqueness.

## Methods

### Study design

This quasi-experimental study was conducted using online training on Covid-19 IPC for healthcare workers in psychiatric institutes from April 2021 to March 2022. First, we developed an online training video about Covid-19 IPC. Then, we recruited voluntary healthcare workers, including doctors, nurses, clinical psychologists, social workers, and medical office workers in psychiatric institutes located in five prefectures in Japan to participate in this training. The participants who agreed to join the training had learned about Covid-19 IPC based on the video and underwent online pre-, post-, and three-month-later-training surveys about knowledge, attitude, and confidence toward Covid-19 IPC. The responses to the surveys were compared to evaluate the efficacy of the training.

### Online training video on Covid-19 infection prevention and control

In this study, we developed an online 30-minute training video on Covid-19 IPC. The training video development committee which developed the online training video comprised psychiatrists, infectious diseases specialists, psychiatric nurses, and managers in psychiatric institutes. The video had guidelines on general IPC, how to put on/take off personal protective equipment (PPE), response to Covid-19 incidence, and infection control in a psychiatric and mental healthcare institute (details are provided in supplement material 1). The components in the video fit the purposes of this study that improve healthcare workers skills and knowledge and get confidence for infection control and stress coping. The video content was validated by an external review committee composed of psychiatrists and staff in health centers. The validity was examined based on guidelines, recommendations by Japanese Ministry of Health, Labour and Welfare, the epidemic situation and feasibility at that time. Based on the validity process by external reviewers, the contents of the training video were elaborated. The training video was provided on the homepage of the Japanese Ministry of Health, Labour and Welfare (the video was provided only in Japanese) (https://www.mhlw.go.jp/stf/seisakunitsuite/bunya/0000121431_00097.html). All participants were asked to review the training video once as an intervention.

### Online survey for the evaluation of the efficacy of the training video

All participants were asked to complete the online survey which included quizzes about knowledge of Covid-19 IPC, questionnaires about confidence and attitudes to infection control, and free comments before, immediately after, and three months after undergoing the training. The reason for the survey after three months from the training was that the healthcare professionals’ skills and knowledge were said to decline after three months of training [22, 23]. The quizzes were multiple choices, and the questionnaires were asked to respond with the seven-point Likert scale. The details of the survey are provided in supplement material 2 (translated from Japanese). The scores in the quizzes and questionnaires were compared between the pre-, post-, and three-month-later surveys to evaluate the short and middle-term efficacy of the training video. Because this was the first study, there was no previous study that validated the quizzes and questionnaires. However, the quizzes and questionnaires were developed by the core members of this study composed of experts in infection control, psychiatry, psychiatric nursing, public health, hospital management, and qualitative study. In addition, the quizzes and questionnaires were contextually validated by outside experts from this study who specialized in similar fields. Since the training video and surveys were provided individually online through their own devices (such as smartphones or computers), almost no cooperation of the participants to complete the questionnaires was required.

Once each participant registered for this study, she/he was provided the link to the study website via email. On the website, each participant provided information about their professional background and responded to the subsequent online survey as pre-evaluation. After completing the pre-evaluation survey, each participant was able to watch the training video online whenever she/he demanded. Each participant could stop and resume watching the training video as she/he liked, but could watch only once. After watching the training video, each participant was requested to start the post-evaluation survey, followed by a three-month-later evaluation survey. Reminder emails were sent automatically if the participant did not complete each survey or watch the training video before the deadline.

### Statistical analyses

The participants’ demographics and occupational experience were summarized. The difference in scores of the quizzes and questionnaires were compared using Wilcoxon signed-rank test. Subgroup analyses were stratified by type of psychiatric institute, type of occupation, age, and experience. Free comments were evaluated qualitatively. All analyses were performed using Stata MP 16.2 in 2022 (STATA Corp., College Station, TX, USA). The statistical significance was set at p ≤ 0.05.

### Sampling and sample size

We approached psychiatric institutes, regardless of the size of the institute, with/without beds or other departments than psychiatry, located in five prefectures in Japan, including Tokyo, Chiba, Aichi, Ehime, and Fukuoka through mental health and welfare centers (governmental organizations established by prefectures) and public health centers (also governmental organizations established by prefectures or cities) in these prefectures. Managers of each psychiatric institute introduced this training video and survey to the healthcare workers in the respective institutes. Participation in this training was completely voluntary and based on the decision of each healthcare worker. The willingness of the participants to undergo training and survey was regarded as agreeing to participate in this study. We calculated the sample size based on the difference of the total scores in quizzes and questionnaires between pre- and three-month-later surveys using the Wilcoxon signed-rank test. We estimated the pre-total scores and three-month-later scores as 0.6 and 0.7, respectively with a common standard deviation of 0.3, using α = 0.05 and β = 0.20. We also assumed approximately 60% of the participants completed all surveys. As a result, 217 participants were required in this study.

### Data collection

All information in this study, including the participants’ background and the responses to the surveys, were obtained through the study website. As to the participants’ backgrounds, all participants provided their expert backgrounds and organizations as long as they did not identify an individual. In the survey composed of quizzes and questionnaires, the responses of the participants were automatically collected online.

### Ethical considerations

The St. Luke’s Ethics Committee Institutional Review Board approved this study (approval number: 21-R061 for the development of video training; 21-R118 for the intervention and surveys). The need for informed consent was waived by the St. Luke’s Ethics Committee Institutional Review Board as the participants voluntarily joined the online training and survey and were deemed to agree to participate in this study.

## Results

A total of 224 participants were included in this quasi-experimental study, out of which 108 (54.0%) were men. The mean age (standard deviation (SD)) of the participants was 47.4 (9.5) years and the mean experience was 18.0 (12.6) years (Table 1). The majority of the study participants were nurses (n = 117, 60.6%), followed by doctors (n = 24, 12.4%), and medical clerks (n = 14, 7.3%). Thirty-two institutes were general hospitals that had other departments also, twenty-three were specialized psychiatric hospitals, and two were psychiatric clinics.

**Table 1:** Participants characteristics

Variables	n = 224
Age, years, mean (standard deviation)	47.4 (9.5)
Male, n (%)	108 (54.0)
Occupation, n (%)	
Nurse	117 (60.6)
Doctor	24 (12.4)
Medical clerk	14 (7.3)
Occupational therapist	12 (6.2)
Pharmacist	6 (3.1)
Mental health worker	5 (2.6)
Clinical psychologist	3 (1.6)
Physical therapist	2 (1.0)
Other healthcare workers	10 (5.2)
Occupational experience, years, mean (standard deviation)	18.0 (12.6)
Location of the psychiatric institute, n (%)	
Aichi	32 (17.8)
Chiba	36 (20.0)
Ehime	57 (31.7)
Fukuoka	30 (16.7)
Tokyo	23 (12.8)
Other	2 (1.2)

**Fig. 1:**
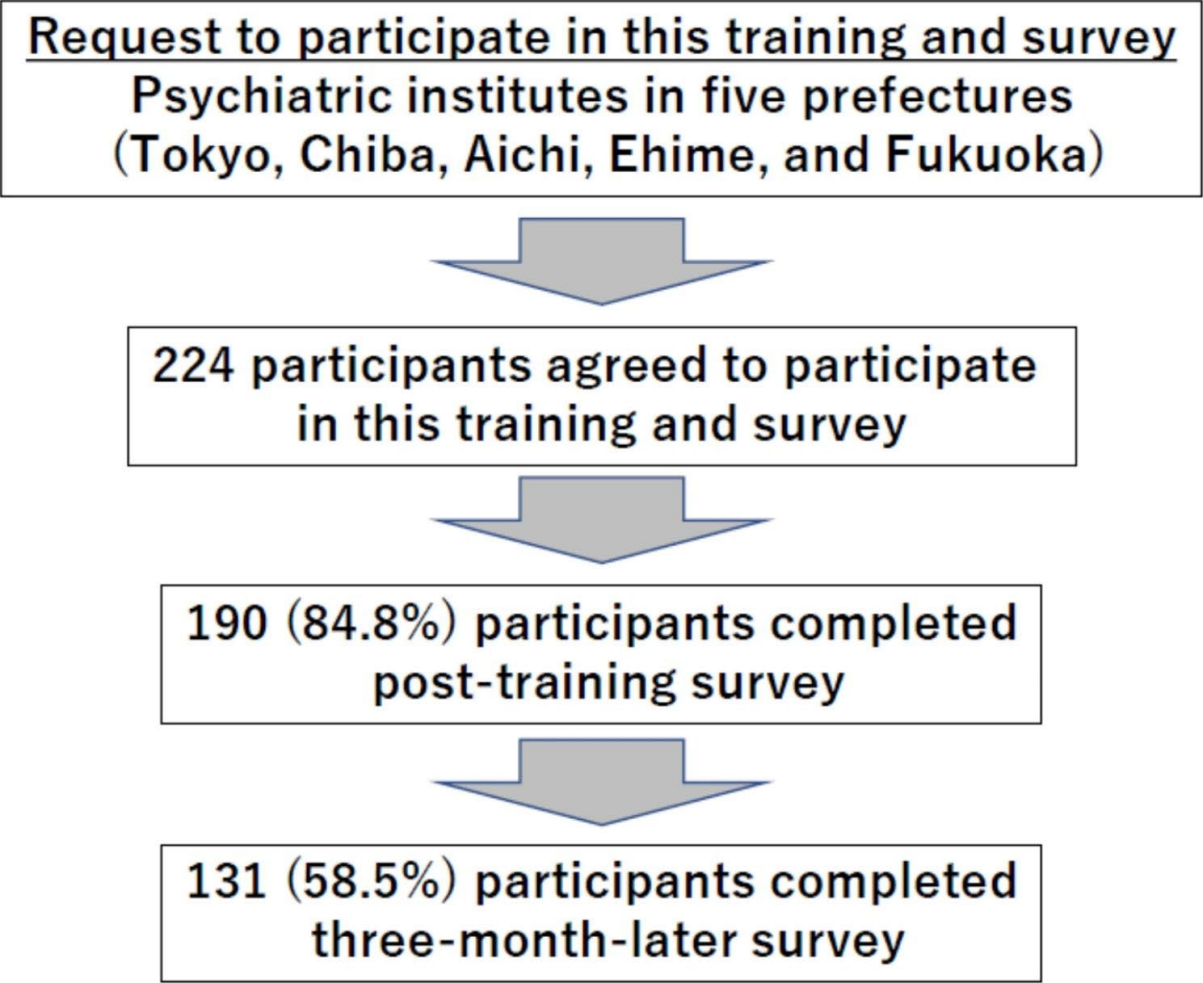
Participants sampling flowchart

Among the participants, 190 (84.8%) completed the post-training survey, and 131 (58.5%) completed a three-month-later survey (Fig. 1). There was no statistical difference in total scores for the quizzes in the pre-training survey between participants who completed all surveys, those who completed the post-training survey, and those who completed only the pre-training survey (57.5 (SD 14.2), 57.2 (SD 14.1), 55.8 (SD 13.3), respectively (p = 0.72). Table 2 shows the correct answer rates to the quizzes in each survey and its comparisons between each survey. The total scores of the post-training (+ 31.1%, SD 15.7, p-value < 0.01) and three-month-later training (+ 14.9%, SD 16.8, p-value < 0.01) surveys showed a significant increase from the pre-training survey scores. In contrast, the total score in the three-month-later survey had significantly decreased from that in the post-training survey (-16.1%, SD 16.7, p-value < 0.01).

**Table 2:** Correct answer rates to the quizzes about knowledge of Covid-19 infection prevention

	Correct answer rate			Between pre- and post-		Between pre- and three-month-later		Between post- and three-month-later	
	Pre-training	Post-training	Three-month later	Change of correct answer rate	p-value	Change of correct answer rate	p-value	Change of correct answer rate	p-value
Quiz a-1	47.8	86.3	74.0	+ 36.8	< 0.01	+ 22.9	< 0.01	-13.7	< 0.01
Quiz a-2	40.2	80.5	52.7	+ 38.9	< 0.01	+ 10.7	0.04	-27.5	< 0.01
Quiz a-3	93.3	97.9	95.4	+ 3.7	0.04	0	1.00	-1.5	0.73
Quiz a-4	46.9	92.6	71.0	+ 47.7	< 0.01	+ 22.9	< 0.01	-19.8	< 0.01
Quiz a-5	81.3	96.3	90.8	+ 14.7	< 0.01	+ 10.7	< 0.01	--5.3	0.12
Quiz a-6	56.7	89.4	77.9	+ 33.2	< 0.01	+ 16.0	< 0.01	-11.5	0.14
Quiz a-7	47.3	92.6	72.5	+ 47.4	< 0.01	+ 31.3	< 0.01	-19.8	< 0.01
Quiz a-8	95.5	98.4	98.5	+ 2.6	0.23	+ 2.3	0.45	-1.5	0.50
Quiz a-9	23.7	76.3	51.1	+ 52.1	< 0.01	+ 25.2	< 0.01	-24.4	< 0.01
Quiz a-10	20.5	74.2	36.6	+ 52.1	< 0.01	+ 18.3	< 0.01	-38.2	< 0.01
Quiz a-11	84.0	94.2	90.8	+ 11.1	< 0.01	+ 9.2	0.03	-5.3	0.12
Quiz a-12	50.4	93.2	72.5	+ 43.7	< 0.01	+ 21.4	< 0.01	-22.9	< 0.01
Quiz a-13	54.0	75.2	58.0	+ 22.6	< 0.01	+ 3.1	0.69	-18.3	< 0.01
Total score, mean (SD)	57.0 (13.9)	88.3 (12.9)	72.5 (14.3)	+ 31.1 (15.7)	< 0.01	+ 14.9 (16.8)	< 0.01	-16.1 (16.7)	< 0.01

Table 3 shows the results of subgroup analyses on the correct answer rates to the quizzes about the knowledge of Covid-19 IPC in each survey and its comparisons between each survey. Participants who belonged to the hospital which had only a psychiatric department had higher total scores in the post-training survey than in the pre-training survey when compared to those who belonged to a general hospital (+ 33.5% vs. +30.6%), although they had similar increases in total scores in the three-month-later survey than the pre-training survey (15.8% vs. 15.5%). Doctors had the highest total scores in the pre-training survey, followed by nurses and others (59.6%, 57.7%, and 55.4%, respectively). However, their scores were comparable in the three-month-later training survey (72.6%, 72.7%, and 72.1%, respectively). As to stratification by age or occupational experience, participants who were younger or had shorter occupational experience had lower total scores in the pre-training survey, but greater increases in the total scores in the post- and three-month-later training surveys.

**Table 3:** Total scores on the quizzes about knowledge of Covid-19 infection prevention and its comparisons by each subgroup

	Total score (SD)			Between pre- and post-		Between pre- and three-month-later		Between post- and three-month-later	
	Pre-training	Post-training	Three-month-later	Change of total score (SD)	p-value	Change of total score (SD)	p-value	Change of total score (SD)	p-value
Type of psychiatric institute									
General hospital (n = 109)	56.2 (13.5)	85.9 (15.0)	71.7 (14.7)	+ 30.6 (17.6)	< 0.01	+ 15.5 (17.0)	< 0.01	-14.6 (18.7)	< 0.01
Psychiatric only (n = 67)	56.9 (12.4)	91.3 (10.6)	74.2 (13.5)	+ 33.5 (13.4)	< 0.01	+ 15.8 (15.2)	< 0.01	-16.6 (12.7)	< 0.01
Type of occupation									
Doctor (n = 24)	59.6 (13.1)	90.1 (14.2)	72.6 (13.8)	+ 30.7 (1.65)	< 0.01	+ 13.0 (17.3)	0.01	-17.8 (18.1)	< 0.01
Nurse (n = 117)	57.7 (12.5)	88.7 (12.3)	72.7 (14.7)	+ 31.2 (15.7)	< 0.01	+ 14.9 (16.4)	< 0.01	-16.2 (17.7)	< 0.01
Other (n = 52)	55.4 (15.8)	87.1 (13.5)	72.1 (14.4)	+ 31.5 (15.7)	< 0.01	+ 15.5 (15.5)	< 0.01	-15.5 (15.2)	< 0.01
Age									
Under 48 years (n = 98)	56.1 (13.8)	87.9 (13.4)	75.9 (13.9)	+ 31.8 (16.5)	< 0.01	+ 18.6 (16.7)	< 0.01	-11.5 (16.7)	< 0.01
48 or older (n = 126)	57.8 (13.9)	88.6 (12.7)	70.3 (14.3)	+ 30.8 (15.1)	< 0.01	+ 12.6 (16.6)	< 0.01	-19.1 (16.1)	< 0.01
Occupational experience									
Less than 20 years (n = 113)	55.8 (14.8)	88.2 (12.7)	74.3 (14.3)	+ 32.2 (15.5)	< 0.01	+ 16.3 (18.0)	< 0.01	-14.8 (16.7)	< 0.01
20 or more (n = 111)	58.3 (12.9)	88.3 (13.3)	70.9 (14.3)	+ 30.3 (15.9)	< 0.01	+ 13.7 (15.7)	< 0.01	-17.4 (16.8)	< 0.01

Table 4 shows the difference in responses to the questionnaire about confidence and attitude toward Covid-19 infection protection. More participants reported having more confidence and less burden towards Covid-19 IPC in the post-training survey compared to the pre-training survey. In the three-month-later survey, more participants reported having more confidence and less burden towards Covid-19 IPC compared to the pre-training survey, but fewer participants did so compared to the post-training survey.

**Table 4:** The change of responses to the questionnaire about confidence and attitude to Covid-19 infection protection

b-1. Which term best describes your weakness or confidence about infection prevention?							
	1. very weak	2. somewhat weak	3. slightly weak	4. neutral	5. slightly confident	6. somewhat confident	7. very confident
Pre-training, n (%)	3 (1.3)	10 (4.5)	47 (21.0)	125 (55.8)	28 (12.5)	8 (3.6)	3 (1.3)
Post-training, n (%)	2 (1.1)	6 (3.2)	21 (11.1)	108 (56.8)	31 (16.3)	19 (10.0)	3 (1.6)
Three-month-later, n (%)	2 (1.5)	1 (0.8)	24 (18.3)	69 (52.7)	21 (16.0)	10 (7.6)	4 (3.1)
b-2. Which term best describes your weakness or confidence about infection prevention of Covid-19?							
	1. very weak	2. somewhat weak	3. slightly weak	4. neutral	5. slightly confident	6. somewhat confident	7. very confident
Pre-training, n (%)	10 (4.5)	40 (17.9)	65 (29.0)	80 (35.7)	20 (8.9)	6 (2.7)	3 (1.3)
Post-training, n (%)	3 (1.6)	14 (7.4)	44 (23.2)	74 (39.0)	39 (20.5)	13 (6.8)	3 (1.6)
Three-month-later, n (%)	3 (2.3)	12 (9.2)	36 (27.5)	53 (40.5)	17 (13.0)	8 (6.1)	2 (1.5)
b-3. Which term best describes your burden to infection prevention of Covid-19?							
	1. None	2. Almost none	3. not much burden	4. neutral	5. little burden	6. somewhat burden	7. very burden
Pre-training, n (%)	1 (0.5)	2 (0.9)	3 (1.3)	23 (10.3)	70 (31.3)	78 (34.8)	47 (21.0)
Post-training, n (%)	1 (0.5)	3 (1.6)	7 (3.7)	32 (16.8)	71 (37.4)	52 (27.4)	24 (12.6)
Three-month-later, n (%)	0 (0.0)	4 (3.1)	1 (0.8)	17 (13.0)	49 (37.4)	44 (33.6)	16 (12.2)
b-4. Which term best describes your opportunities to learn about infection prevention strategies against Covid-19?							
	1. none	2. almost none	3. not much	4. once a month	5. often	6. very often	7. more than once a week
Pre-training, n (%)	0 (0.0)	6 (2.7)	86 (38.4)	50 (22.3)	57 (25.5)	20 (8.9)	5 (2.2)
Post-training, n (%)	0 (0.0)	7 (3.7)	68 (35.8)	44 (23.2)	52 (27.4)	16 (8.4)	3 (1.6)
Three-month-later, n (%)	0 (0.0)	2 (1.5)	42 (32.1)	29 (22.1)	38 (29.0)	13 (9.9)	7 (5.3)
b-5. Which term best describes how aggressively your institute is working on preventing Covid-19 infection?							
	1. very	2. somewhat	3. a little	4. neutral	5. not much	6. almost none	7. none
Pre-training, n (%)	11 (4.9)	56 (25.0)	79 (35.3)	67 (29.9)	11 (4.9)	0 (0.0)	0 (0.0)
Post-training, n (%)	4 (2.1)	44 (23.2)	71 (37.4)	59 (31.1)	10 (5.3)	1 (0.5)	1 (0.5)
Three-month-later, n (%)	7 (5.3)	31 (23.7)	49 (37.4)	35 (26.7)	7 (5.3)	2 (1.5)	0 (0.0)
b-6. Which term best describes your confidence to put on/take off personal protective equipment, and to teach how to do for others?							
	1. very weak	2. somewhat weak	3. slightly weak	4. neutral	5. slightly confident	6. somewhat confident	7. very confident
Pre-training, n (%)	13 (5.8)	19 (8.5)	49 (21.9)	58 (25.9)	48 (21.4)	23 (10.3)	14 (6.3)
Post-training, n (%)	2 (1.1)	7 (3.7)	31 (16.3)	68 (35.8)	48 (25.3)	23 (12.1)	11 (5.8)
Three-month-later, n (%)	2 (1.5)	8 (6.1)	30 (22.9)	40 (30.5)	32 (24.4)	10 (7.6)	9 (6.9)
b-7. Which term best describes your confidence in your action as both an individual and organization when a cluster has occurred?							
	1. very weak	2. somewhat weak	3. slightly weak	4. neutral	5. slightly confident	6. somewhat confident	7. very confident
Pre-training, n (%)	0 (0.0)	13 (5.8)	50 (22.3)	69 (30.8)	68 (30.4)	21 (9.4)	3 (1.3)
Post-training, n (%)	0 (0.0)	6 (3.2)	25 (13.2)	59 (31.1)	67 (35.3)	26 (13.7)	7 (3.7)
Three-month-later, n (%)	0 (0.0)	1 (0.8)	3 (2.3)	25 (19.1)	40 (30.5)	40 (30.5)	15 (11.5)

The analysis of the free comments showed that the participants expressed appreciation for the confirmation of their knowledge and skills. However, few participants expressed anger at the excessive measures taken, which was consistent with the results of the analysis of quantitative data from the three-month-later survey.

## Discussion

This quasi-experimental study demonstrated that healthcare workers had advanced their knowledge and confidence in the IPC for Covid-19 by undergoing 30 min of online training. Although each healthcare institute may provide institutional training on Covid-19 IPC for their healthcare workers either in person or online, our study proved its efficacy objectively. In addition, to the best of our knowledge, this is the first study to develop an online IPC training module on Covid-19 for psychiatric healthcare workers and evaluate its efficacy.

Based on quizzes about knowledge in IPC and the difference in the correct answer rates, we could hypothesize the characteristics and problems in IPC for psychiatric healthcare workers. First, the correct answer rates were lowest in the quiz about patient care during cluster and zoning in the pre-training survey, resulting in the greatest increase in survey scores in the post-training survey. Since the pre-training quiz focused on psychiatric patient care, rather than general patient care, IPC in psychiatric situations seemed to be difficult and required additional education. In contrast, the quiz about zoning was a common strategy of IPC both in psychiatric and general situations, suggesting zoning itself was a complicated issue to be understood. The highest correct answer rates were observed in quizzes 3 and 8, which were rated to avoid closed space or close contact, in the pre-training survey. The possible reason for such high scores might be due to the extensive campaign by the Japanese Ministry of Health Labour and Welfare. The Ministry has repeatedly advocated for the citizens to avoid the three Cs; “closed spaces with poor ventilation,“ “crowded spaces with many people nearby,“ and “close-contact settings such as close-range conversations,“ [24] and it would have permeated them. Based on this study’s findings, it could be inferred that psychiatric healthcare workers could advance their knowledge about psychiatric IPC by training, even though they had limited knowledge about psychiatric IPC compared to general IPC. More psychiatric-specific training would improve psychiatric IPC in clinical practice.

The confidence and aggressive attitude towards IPC for Covid-19 improved from pre-training to post- and three-month-later training based on our survey. Although the confidence and aggressive attitude may be due to the increased time of exposure to the Covid-19 pandemic, our training still could have contributed. The fact that correct answer rates increased from pre- to post-training but decreased from post- to three-month-later in our quiz proves that our training improved participants’ knowledge from pre- to post-training, but its effect weakened as time passed. Similar effects could be attributed to the increase in confidence and aggressive attitude. Apart from confidence and attitude, the burden of IPC for Covid-19 was reported to have increased in the three-month-later training survey compared to the pre- and post-training survey which could be due to the emergence of the first Omicron variant case in November 2021 in Japan and the subsequent rapid increase in Omicron variants from December 2021 [25]. In fact, the incidence of Covid-19, especially the Omicron variant, dramatically increased from January 2022 in Japan, [26] when the three-month-later training survey was being conducted (January 2022). In summary, our online training could have also contributed to improving the confidence and aggressive attitude towards IPC for Covid-19 among psychiatric healthcare workers.

Compared to previous studies, our study accumulated some additional evidence of IPC training for Covid-19. All healthcare institutes in this study were psychiatric institutes, whereas other studies were conducted in general hospitals or hospitals in a certain community. Regarding the evaluation of the effectiveness of the IPC training, our study used original evaluation and self-reported surveys like other previous studies [12, 13]. These evaluations themselves, including those in our study, are varied and not well-validated; therefore, we could not compare the effectiveness of IPC training between studies. Universal and well-validated evaluation methods are required for further studies. In addition, it may be better to evaluate the incidence of Covid-19 or related mortality in the healthcare institutes as the effectiveness of the IPC training, although it would be very challenging due to complicated circumstances, such as pandemic periods, the uniqueness of the institutes, and the vaccination status.

Our study has some limitations. First, not all participants had completed the three-month-later survey, suggesting that we could not assume the effects of our online training among them; causing the non-responder bias. However, the scores of quizzes about knowledge about IPC for Covid-19 in the pre-training survey were similar among those who completed all surveys, those who did until the post-training survey, and those who did only the pre-training survey. Therefore, similar learning effects might be expected in those who had dropped out after completing the online training. Second, our training may not be generalized to all psychiatric healthcare workers in other countries, because some issues were specific to the Japanese situation. In addition, because our online training was provided only in Japanese, it could not be reviewed and evaluated by the non-Japanese population. The modification of our training according to the prevailing situations would be useful to improve the knowledge and confidence of IPC for Covid-19 in each country. Third, because we evaluated participants’ knowledge at a maximum of three months post-training, the long-term effects of the training were uncertain. However, repeating the training course might be useful to improve and sustain the knowledge among psychiatric healthcare workers. Fourth, although our quizzes and questionnaires were contextually validated, there was no guarantee for validity. Further studies are required to validate our quizzes and questionnaires. Finally, there may be unmeasured confounders in our study. For example, some healthcare workers may experience covid-19 clusters in their psychiatric institutes during the study period. They may obtain more skills and knowledge from the cluster experiences than from our online training.

## Conclusion

Thirty minutes of online training about IPC for Covid-19 improved the knowledge, confidence, and attitude among psychiatric healthcare workers. Regular online training would help in preventing the transmission or formation of clusters of Covid-19 in psychiatric healthcare institutes.

## Electronic supplementary material

Below is the link to the electronic supplementary material.

Supplementary Material 1

Supplementary Material 2

## Data Availability

The datasets generated and/or analyzed during the current study are not publicly available as the St. Luke’s Ethics Committee Institutional Review Board does not allow to do so; however, they are available from the corresponding author on reasonable request and upon the board approval.
