# Supplementary material for: Online training of Covid-19 infection prevention and control for healthcare workers in psychiatric institutes
Source: BMC Psychiatry. 2023 May 9;23:325. doi: 10.1186/s12888-023-04826-5 (PMC10169098; doi:10.1186/s12888-023-04826-5)
Supplement: Supplementary file 1 — Supplementary Material 1 [file 12888_2023_4826_MOESM1_ESM.docx]

Supplement material 1. Components of the online training video

- General infection control prevention

Avoiding three Cs

Closed spaces, crowded places, close-contact settings

Regular ventilation

Wearing mask

Surgical mask

Hand sanitization

　 Alcohol hand sanitization when enter/go out room, open/ close door, before and after procedures

Self-healthcare

　Check body temperature and symptoms

Important points when eating

　 Eat in silence

　 Do not sit face to face

Infection control in each situation

Infection control for all pathogens

Standard precaution

Infection control for Covid-19

Wearing mask for droplet infection control

Putting on gloves for contact infection control

Wearing N95 mask for aerosol infection control

- How to put on/take off personal protective equipment

Hand sanitization before put on personal protective equipment

Covering nose and mouth completely by mask

Putting on gloves after wearing gown and mask

Pay attention not to be contaminated when taking gloves off

Change gloves for each procedure or patient

Do not touch eyes, nose and mouth

- Response to Covid-19 incidence

First action

Contact with public health centers

Identify Covid-19 patients and close contact people

Check symptoms of Covid-19 patients

Information sharing for staffs

Start infection control

Zoning

Red, yellow, and green zone

Preparation for the incidence

Reconfirmation for general infection control

Check infection control manuals in the hospital

Preparation for putting on/taking off personal protective equipment

Simulation for covid-19 incidence

Prior consultation to governmental organizations

- Infection control in a psychiatric and mental healthcare institute
- Infection control and mental health care

Approaches to stress reduction for the staffs

Self-care

Line-care
