# Supplementary material for: Online training of Covid-19 infection prevention and control for healthcare workers in psychiatric institutes
Source: BMC Psychiatry. 2023 May 9;23:325. doi: 10.1186/s12888-023-04826-5 (PMC10169098; doi:10.1186/s12888-023-04826-5)
Supplement: Supplementary file 2 — Supplementary Material 2 [file 12888_2023_4826_MOESM2_ESM.docx]

**Supplement material 2:**

Quizzes about knowledge of Covid-19 IPC and questionnaires about confidence and attitudes towards infection control

Quizzes

a-1. Which are the useful approaches for Covid-19 infection control in daily life?

Avoiding three Cs; "closed spaces with poor ventilation," "crowded spaces with many people nearby," and "close-contact settings such as close-range conversations"

Routine room ventilation

Alcohol disinfection without washing hands if hands are contaminated

Measuring body temperature every day

Eating meals while chatting

a-2. Which are the effective approaches for infection control based on the infection route?

Wearing gloves to prevent contact infection

Wearing an N95 mask to prevent droplet infection

Wearing a surgical mask to prevent airborne (aerosol) infection

Wearing personal protective equipment (gown or apron) to prevent contact infection

a-3. You provide care for the patient who is suspected to have Covid-19 infection. Which one is not recommended?

Disinfecting thermometer with alcohol

Care for the patient in a poorly ventilated and closed room

Ask the patient to wear the surgical mask

Assess the mental status of the patient

a-4. Which one must be worn last when you wear personal protective equipment?

Gloves

Mask

Gown

Face shield

Cap

a-5. Which are the correct approaches to wearing or taking off personal protective equipment?

Sanitize hands before wearing personal protective equipment

Cover nose and mouth completely with a mask

Pay attention not to contaminate your hands when removing gloves

Use the same gloves until your shift is over

a-6. Which are the correct choices to prepare for the increased incidence of Covid-19 infections?

Make sure general infection control

Make sure nosocomial infection control manual

Simulation of the Covid-19 incidence

Training in putting on/taking off the personal protective equipment

Prior consultation with the government

a-7. What is the first action to be taken when a patient is diagnosed with Covid-19 infection in a psychiatric institute?

Start zoning in the ward

Transfer personal protective equipment to the institute

Modify shifts of the staffs

Share information with the staff

a-8. Which is the most effective way to prevent the spread of infection during the Covid-19 infection cluster in a psychiatric institute?

Clean rooms, provide meals and do laundry as usual

Entrust infection control to the governmental health center

Save sanitizers

Pay attention to infection control even in the staff break rooms

a-9. Which are the correct approaches for psychiatric patients when a Covid-19 cluster has occurred?

Provide infection control adjusted for psychiatric patients

Explain the results of laboratory tests to patients

Pay attention to patients’ physical status

Provide sanitizers to all psychiatric patients to be used anytime

a-10. Which is the correct zoning information?

Not necessary to wear gloves in a red zone

Take personal protective equipment off in a yellow zone

Wear a surgical mask even in a green zone

Put contaminated waste boxes in a red zone

It is important not to spread infection from the yellow zone

a-11. What should be disinfected when a Covid-19 cluster has occurred

Bed rails

Doorknobs

Vending machines

Sphygmomanometer

Keys to psychiatric wards

a-12. Which are the correct approaches when a Covid-19 cluster has occurred?

Establish a task force for infection control

Modify staff shifts

Obtain enough personal protective equipment

Measure body temperature only once a day for close-contact patients

Modify tasks for room cleaning, providing meals, and laundry

a-13. Which are the unique characteristics of infection control in psychiatric institutes?

Many doors with lock

A limited number of infection control specialists

Limited opportunities to contact multidisciplinary

A limited number of reports about physical symptoms from psychiatric patients

More independent from local healthcare centers

Questionnaires

b-1. Which term best describes your weakness or confidence about infection prevention?

b-2. Which term best describes your weakness or confidence about infection prevention of Covid-19?

b-3. Which term best describes your burden to infection prevention of Covid-19?

b-4. Which term best describes your opportunities to learn about infection prevention strategies against Covid-19?

b-5. Which term best describes how aggressively your institute is working on preventing Covid-19 infection?

b-6. Which term best describes your confidence to put on/take off personal protective equipment, and to teach how to do for others?

b-7. Which term best describes your confidence in your action as both an individual and organization when a cluster has occurred?
